# BAC-End Sequence-Based SNP Mining in Allotetraploid Cotton (*Gossypium*) Utilizing Resequencing Data, Phylogenetic Inferences, and Perspectives for Genetic Mapping

**DOI:** 10.1534/g3.115.017749

**Published:** 2015-04-09

**Authors:** Amanda M. Hulse-Kemp, Hamid Ashrafi, Kevin Stoffel, Xiuting Zheng, Christopher A. Saski, Brian E. Scheffler, David D. Fang, Z. Jeffrey Chen, Allen Van Deynze, David M. Stelly

**Affiliations:** * Department of Soil and Crop Sciences, Texas A&M University, College Station, Texas 77843; † Interdisciplinary Genetics Program, Texas A&M University, College Station, Texas 77843; ‡ Seed Biotechnology Center, University of California, Davis, California 95616; § Clemson University Genomics Institute, Clemson University, Clemson, South Carolina 29634; ** USDA-ARS, Genomics and Bioinformatics Research Unit, Stoneville, Mississippi 38766; †† USDA-ARS, Cotton Fiber Bioscience Research Unit, New Orleans, Louisiana 70124; ‡‡ Department of Molecular Biosciences, Center for Computational Biology and Bioinformatics, and Institute for Cellular and Molecular Biology, The University of Texas, Austin, Texas 78712

**Keywords:** SNP genotyping, cotton genomics, resequencing, BAC-derived SNPs, intraspecific

## Abstract

A bacterial artificial chromosome library and BAC-end sequences for cultivated cotton (*Gossypium hirsutum* L.) have recently been developed. This report presents genome-wide single nucleotide polymorphism (SNP) mining utilizing resequencing data with BAC-end sequences as a reference by alignment of 12 *G. hirsutum* L. lines, one *G. barbadense* L. line, and one *G. longicalyx* Hutch and Lee line. A total of 132,262 intraspecific SNPs have been developed for *G. hirsutum*, whereas 223,138 and 470,631 interspecific SNPs have been developed for *G. barbadense* and *G. longicalyx*, respectively. Using a set of interspecific SNPs, 11 randomly selected and 77 SNPs that are putatively associated with the homeologous chromosome pair 12 and 26, we mapped 77 SNPs into two linkage groups representing these chromosomes, spanning a total of 236.2 cM in an interspecific F2 population (*G. barbadense* 3-79 × *G. hirsutum* TM-1). The mapping results validated the approach for reliably producing large numbers of both intraspecific and interspecific SNPs aligned to BAC-ends. This will allow for future construction of high-density integrated physical and genetic maps for cotton and other complex polyploid genomes. The methods developed will allow for future *Gossypium* resequencing data to be automatically genotyped for identified SNPs along the BAC-end sequence reference for anchoring sequence assemblies and comparative studies.

Marker development in crop species has been an important aspect to facilitate genomics-based crop improvement. Single nucleotide polymorphisms (SNPs) are the most abundant form of markers in all organisms, because they have a possibility of occurring at every nucleotide position in the genome. This makes them ideal candidates for construction of high-density genetic maps, which can then be used for marker-based crop improvement and genetic analyses. In general, for marker-assisted selection, a large number of molecular markers are required in crop species as the majority of the traits of interest such as yield, drought and heat tolerance, nitrogen and water use efficiency, disease resistance, and fiber quality are complex and controlled by many genomic loci of small effect. Therefore, for marker-assisted breeding to be effective, markers for most of the regions controlling a trait need to be included in selection criteria (Mammadov *et al.* 2012). SNPs have been found to occur approximately every 60–120 bp in the maize genome (Ching *et al.* 2002), every 268 bp in the rice genome (Shen *et al.* 2004), and every 185–266 bp in the soybean genome (Lam *et al.* 2010). The markers developed to date in cotton have been limited mostly to amplified fragment length polymorphisms (AFLPs), restriction fragment length polymorphisms (RFLPs), and simple sequence repeats (SSRs). Some recent efforts to identify SNPs in cotton using transcriptome sequencing in intraspecific and interspecific cotton lines (Hulse-Kemp *et al.* 2014; Zhu *et al.* 2014; Ashrafi *et al.* 2015), single copy sequences between *G. hirsutum* and *G. barbadense* (Van Deynze *et al.* 2009), and a variety of reduced representation libraries (RRLs) techniques investigating combinations of *G. hirsutum* lines (Byers *et al.* 2012; Rai *et al.* 2013; Zhu *et al.* 2014; Islam *et al.* 2015; Gore *et al.* 2014) have been reported and have been increasing in efficiency using a diverse range of germplasm. These studies of cotton have generated anywhere from a few hundred SNPs to tens of thousands of SNPs. However, success with cotton has been limited compared to crop species such as maize (Elshire *et al.* 2011; Hansey *et al.* 2012), soybean (Hyten *et al.* 2010), barley, and wheat (Poland *et al.* 2012), which also used a broad range of germplasm and have been able to develop hundreds of thousands of SNPs.

Reasons for reduced success with SNP development within *Gossypium* compared to efforts in other crops are perhaps due to the evolutionary history of the cotton genome and recent polyploid generation. The most widely cultivated species of cotton, *Gossypium hirsutum* L., descends from a recently formed allotetraploid hybrid (1–2 MYA), 2n = 4x = 52, genomic equation 2[AD]_1_ (Wendel *et al.* 2009). Modern cultivated cotton, including Upland types, have undergone at least two independent bottleneck events, domestication, and elite cultivar selection, which further reduced the overall diversity that can be found in elite cultivars. The ancestral A- and D-like genomes are thought to have diverged only 5–10 MYA. Because of limited time for evolutionary divergence, homeologous regions of the A- and D-subgenomes of cotton retain a very high similarity. Although reference sequences have recently become available for the diploid cottons, *G. raimondii* (extant relative to the allotetraploid D-subgenome) (Paterson *et al.* 2012), and *G. arboreum* (extant relative to the allotetraploid A-subgenome) (Li *et al.* 2014), a reference is not yet available for allotetraploid *G. hirsutum*.

Large-scale SNP development in cotton has been hindered by the lack of a high-quality *G. hirsutum* reference which, in concert with high levels of similarity between subgenomes and low levels of diversity, makes it very difficult to unambiguously map short-read sequences obtained from next-generation sequencing technologies (Zhu *et al.* 2014). Recently, we developed three independent bacteria artificial chromosome (BAC) libraries, two generated from restriction-enzyme partial digestion (BstYI/HindIII) and one generated by random-shearing (C. A. Saski, A. M. Hulse-Kemp, J. Schmutz, B. Liu, D. M. Stelly, J. A. Scheffler, D. C. Jones, D. G. Peterson, Z. J. Chen, and B. E. Scheffler). From these, 179,209 high-quality BAC-end sequences (BESs) were generated. Development of markers aligned to BAC-end sequences is desired for several reasons. First, the quality of Sanger-sequencing is currently the highest on a single read basis. Second, the markers can serve as a rigid interface between a BAC-based physical map with genetic maps. Third, taken together, long-range contiguity and marker distribution can begin to be contextualized on a genome-wide scale. Recent reports suggest the utility of BESs in simple sequence repeat (SSR) marker development in pigeon pea (Bohra *et al.* 2011), cotton (Frelichowski *et al.* 2006), and peanut (Wang *et al.* 2012), and for SNP development via PCR-directed sequencing methods in apple (Han *et al.* 2009) and in *Citrus* (Ollitrault *et al.* 2012) to anchor genomic sequences.

Thus, the primary goals of the present study were: to develop large numbers of genomic-based SNPs for cultivated cotton, *G. hirsutum*; to compare the levels of diversity among elite cultivars, a wild accession, an additional tetraploid species (*G. barbadense* L.), and a diploid cotton species (*G. longicalyx*); to experimentally validate *in silico*–derived SNPs; and to demonstrate mapping ability and utility of developed SNPs. This article reports on utilizing BESs as a high-quality reference for SNP discovery through resequencing. The new SNPs will be a resource for SNP-based integration of the physical and genetic maps, and the methodology can also serve as a useful model in resolving other complex plant genomes.

## Materials and Methods

### Source of BAC clones and BAC-end sequencing

Two complementary BAC libraries (BstYI and HindIII) and a random sheared BAC library from *G. hirsutum* genetic standard line Texas Marker-1 (TM-1) were used in this study. BAC DNA was isolated and sequenced by Sanger methods at USDA-ARS (Stoneville, MS). A total of 179,209 BAC-end sequences were retained after quality trimming and filtering (LIBGSS_039228) (Saski *et al.* unpublished data).

### Plant material and DNA extraction

The seed of 11 *G. hirsutum* lines (TM-1, Sealand 542, PD-1, Paymaster HS-26, M-240 RNR, Fibermax 832, Coker 312, SureGrow 747, Stoneville 474, Tamcot Sphinx, and TX0231) and the *G. barbadense* genetic standard line, 3-79, were planted at Texas A&M University, University of California Davis, or the USDA-ARS facility in New Orleans, Louisiana. Young leaf tissues were sampled from each plant and used to isolate genomic DNA using the Qiagen DNeasy (Qiagen, Valencia, USA) plant extraction kit following manufacturer instructions, including RNase digestion. DNA concentrations and qualitative absorbance values were determined using the Nanodrop Spectrophotometer (Thermo Fisher Scientific, Waltham, MA).

### Library preparation and sequencing

Double-stranded DNA was quantified with a PicoGreen assay on the Synergy HT plate reader (Bio-Tek, Highland Park, IL). Libraries were prepared using an in-house protocol with individual barcoding. One and a half micrograms of each DNA sample was randomly sheared using a Bioruptor instrument (Diagenode, Denville, NJ), and then size selection was performed using AMPure XP beads to 300–500 bp. Fragments were end-repaired using NEBNext End Repair Module and then purified with AMPure beads. Next, an adenine was ligated at the end of the fragments, adapters were ligated, and the final products were purified with AMPure beads. Enrichment PCR was performed for 14 cycles, and then the PCR product was run on a 1.5% gel to confirm enrichment of product and size range. A final round of purification was performed using AMPure beads. The final libraries were assessed for quality with the Bioanalyzer (Agilent, Santa Clara, CA) to determine final library size and concentration. Each sample was sequenced with paired-end sequencing (2×100 bp) on two Illumina HiSeq2500 lanes. Raw, paired-read sequence files were uploaded to NCBI under BioProject PRJNA257223 and SRA numbers (SRX667500, *G. hirsutum* TM-1; SRX668168, *G. hirsutum* Sealand 542; SRX668322, *G. hirsutum* PD-1; SRX668354, *G. hirsutum* Paymaster HS-26; SRX669467, *G. hirsutum* M-240 RNR; SRX669468, *G. hirsutum* Fibermax 832,; SRX669469, *G. hirsutum* Coker 312; SRX669470, *G. hirsutum* SureGrow 747; SRX669471, *G. hirsutum* Stoneville 474; SRX669472, *G. hirsutum* Tamcot Sphinx; SRX669473, *G. hirsutum* TX0231; SRX669474, *G. barbadense* 3-79). Raw reads for *G. hirsutum* Acala Maxxa and for *G. longicalyx* were obtained from the NCBI Small Read Archive under numbers SRR617482 and SRR617704.

### SNP mining from *G. hirsutum* aligned to BAC-end sequences

All raw sequence files were assessed for initial quality using FastQC (http://www.bioinformatics.babraham.ac.uk/projects/fastqc/), and the first 13 bases from each read were removed due to poor quality. The remaining reads were quality-trimmed, and adapters and any reads fewer than 40 bases were removed using the FastX toolkit (http://hannonlab.cshl.edu/fastx_toolkit/). The files were assessed for final quality and concatenated into a single file to be utilized as single-end read data due to the size of the BESs being used as a reference. Once the sequences had been processed, they were imported into CLC Genomics Workbench v 6.0.2 (Valencia, CA). Reads from Sealand 542, PD-1, and 3-79 were aligned to the BAC-end sequence reference using different length and similarity fractions to determine optimal parameters. Iteration-1 utilized length fraction 0.70 and similarity fraction 0.99, whereas Iteration-2 utilized length fraction 0.99 and similarity fraction 0.98. SNPs were called using the Probabilistic variant caller in CLC Genomics Workbench using a minimum sequence depth of six, variant probability of 50.0, required presence in both forward and reverse reads, four maximum expected variants, and one standard genetic code. Theoretical homeo-SNP positions (variants between homeologous regions caused by ambiguous mapping of homeologous reads from different subgenomes in the allotetraploid) were determined by aligning the TM-1 Illumina-based sample, which is the same genotype as the reference, to call SNPs using the same parameters as all other samples. These identified homeo-SNP positions were removed from the SNPs identified in the other samples.

A random set of called 32 SNP positions was manually viewed to assess quality of alignments around the SNP positions, which included markers only found in Iteration-1 and markers only found in Iteration-2; markers found in both Iteration-1 and Iteration-2 from the SNPs discovered using *G. barbadense* 3-79 were selected for empirical testing. Half of the 32 markers were identified as overlapping transcriptome-derived SNPs as reported in Hulse-Kemp *et al.* (2014), whereas half of the SNPs did not overlap the Hulse-Kemp *et al.* dataset and thus they were assumed to not be associated with the transcriptome. Primers were designed for KASP (LGC Genomics) SNP assays using BatchPrimer3 (allele-specific primers and allele-flanking primers; Tm: optimum, 57°; minimum, 55°; maximum, 60°; max difference, 2°; product size: minimum, 50 bp; optimum, 50 bp; maximum, 100 bp). Primers were synthesized and diluted according to KASP developer (LGC Genomics, Hoddesdon, UK) instructions. KASP assays were run on a “*G. barbadense* screening panel” (Supporting Information, Table S1) containing 12 samples including TM-1 (Stelly Lab), TM-1 (USDA), 3-79 (×2), F1 – 3-79×TM-1 (×2), RIL01-04 (3-79×TM-1), and water nontemplate control (×2) according to the manufacturer’s suggested PCR conditions. Plates were read using the Pherastar at 38, 44, and 50 cycles, and then they were analyzed using the KlusterCaller program. SNP assays were labeled as “good” if samples produced clean clusters that allowed for differentiation and scoring of the two parents and the F1 genotypes, or as “bad” if no definable clusters were produced or no amplification occurred. (These definitions of “good” and “bad” are used subsequently throughout the rest of the article.) Markers included in the “good” and “bad” categories for Iteration-1 and Iteration-2 were calculated (Table 1) to determine optimal parameter settings.

**Table 1:** KASP assay screening results for *G. barbadense*–derived markers

Type	Marker Name	KASP Result	Identified in *G. barbadense* Mapping		Homeo-SNP Removal		Final Result	
			Iteration 1	Iteration 2	Iteration 1	Iteration 2	Iteration 1	Iteration 2
Not Transcriptome-Associated	GH_TBb001A07f_381	Good	Yes	Yes	Retain	Retain	Good	Good
	GH_TBb001A07f_486	Bad	No	Yes	—	Remove	—	—
	GH_TBb001A23r_531	Bad	No	Yes	—	Retain	—	Bad
	GH_TBb001A23r_614	Good	Yes	Yes	Retain	Retain	Good	Good
	GH_TBb001D22r_204	Good	Yes	Yes	Retain	Retain	Good	Good
	GH_TBb001D22r_445	Bad	Yes	No	Retain	—	Bad	—
	GH_TBb001D22r_511	Bad	Yes	Yes	Retain	Retain	Bad	Bad
	GH_TBb001B05f_180	Good	Yes	Yes	Remove	Remove	—	—
	GH_TBb001B05f_564	Bad	Yes	Yes	Remove	Remove	—	—
	GH_TBb001C03f_116	Good	No	Yes	—	Retain	—	Good
	GH_TBb001C03f_401	Good	Yes	Yes	Retain	Retain	Good	Good
	GH_TBb001F01r_117	Bad	Yes	Yes	Remove	Remove	—	—
	GH_TBb001F01r_310	Bad	Yes	No	Remove	—	—	—
	GH_TBb001A17r_218	Bad	Yes	Yes	Remove	Remove	—	—
	GH_TBb001F06f_303	Bad	No	Yes	—	Remove	—	—
							
Transcriptome-Associated	GH_TBb004J20r_76	Good	Yes	Yes	Remove	Remove	—	—
	GH_TBb004J20r_348	Good	Yes	Yes	Retain	Retain	Good	Good
	GH_TBb053N14f_270	Good	Yes	Yes	Retain	Retain	Good	Good
	Gh_TBh036B20r_583	Good	No	Yes	—	Retain	—	Good
	GH_TBr162H20f_547	Bad	Yes	Yes	Remove	Remove	—	—
	GH_TBb046O02r_64	Bad	Yes	Yes	Remove	Remove	—	—
	GH_TBb046O02r_138	Bad	Yes	Yes	Remove	Remove	—	—
	GH_TBb046O02r_418	Bad	Yes	Yes	Remove	Remove	—	—
	GH_TBb069A06f_248	Bad	Yes	No	Retain	—	Bad	—
	GH_TBb069A06f_304	Bad	Yes	Yes	Remove	Remove	—	—
	GH_TBh034D07r_276	Good	Yes	Yes	Retain	Retain	Good	Good
	GH_TBh030P12r_332	Bad	Yes	Yes	Remove	Remove	—	—
	GH_TBb119O19f_465	Maybe (DOM)	Yes	Yes	Retain	Retain	N/A	N/A
	GH_TBh055K17f_57	Bad	Yes	Yes	Remove	Remove	—	—
	GH_TBh055K17f_506	Bad	Yes	Yes	Remove	Remove	—	—
	GH_TBh023O21f_162	Good	Yes	No	Remove	Retain	—	Good
	GH_TBh023O21f_537	Good	Yes	Yes	Retain	Retain	Good	Good

KASP assay screening results for *G. barbadense*–derived markers for mapping parameter optimization in CLC Genomics Workbench. Iteration-1 was performed using the following mapping parameters: 0.70 length fraction and 0.99 similarity fraction. Iteration-2 was performed using the following mapping parameters: 0.99 length fraction and 0.98 similarity fraction.

All 12 *G. hirsutum* samples and the *G. barbadense* sample were analyzed using Iteration-2 parameters. The *G. longicalyx* sample was analyzed using the same length fraction but similarity fraction of 0.96. Samples were processed individually and homeo-SNPs identified using TM-1 were removed. Unique SNP positions were collated into a master file and required 100% homozygous identification in at least one *G. hirsutum* line. Positions where coverage was 1 to 2 SDs above the average coverage in the homozygous sample(s) were removed, because regions with high coverage are likely to be associated with repetitive regions. Additional unique SNP positions identified as being homozygous in *G. barbadense* or *G. longicalyx* were added to the master file. Only positions with probability scores >0.98 were retained for *G. barbadense* and *G. longicalyx*. A hierarchical method was utilized to determine retention; SNPs were retained in the *G. hirsutum* file initially, then in *G. barbadense*, and then in *G. longicalyx*. Three nonredundant variant call format (VCF) files were obtained from each of the three species groups. These VCF files were uploaded to dbSNP in NCBI (batch 1062135 for *G. hirsutum* lines, batch 1062133 for *G. barbadense*, and batch 1062134 for *G. longicalyx*).

A final VCF file was derived using GATK software (https://www.broadinstitute.org/gatk/) that contained genotypes at all positions for all samples using the list of nonredundant SNP positions, binary alignment map (BAM) files exported from CLC Workbench for each individual sample, and BES FASTA file. The GATK UnifiedGenotyper command with default settings except for the GENOTYPE_GIVEN_ALLELES, EMIT_ALL_SITES and minimum base quality of 20 options were used to call genotypes for all previously identified SNP positions along the BES reference for the 12 *G. hirsutum* lines, *G. barbadense*, and *G. longicalyx*. Distributions of SNP types, number of missing calls per sample, and number of heterozygous calls per sample were calculated from the final VCF file using VCFtools (http://vcftools.sourceforge.net/). The number of homozygous differences between samples was determined using BCFtools command gtcheck.

### BAC-end sequence and BES-derived SNP annotation

A total of 179,209 BAC-end sequences that were used as the reference were screened for repetitive content with the RepeatMasker software (Tarailo-Graovac and Chen 2009) version open-2.0.5 (database version 20140131) and cross match version 1.090518 (distributed with the Consed software) (Gordon *et al.* 1998), with viridiplantae as the query species. Repeat masked sequences were aligned using blastx (Altschul *et al.* 1990) to the nonredundant protein database (Genbank) using an e-value cutoff of 1e^−5^ and the output was filtered for best hit using an in-house .xml parser to annotate BESs. All identified SNPs were analyzed to determine if they were located in BESs associated with coding regions.

### Marker alignment to diploid cotton reference genomes

Markers identified were exported with 50-bp flanking sequence on both sides. SNPs were coded with IUPAC ambiguity codes and the total 101 bp sequence of each SNP with flanking sequence was mapped using Burrows-Wheeler Aligner (BWA) in Galaxy (Goecks *et al.* 2010) using default settings. All SNPs were mapped to both the *Gossypium raimondii* (D_5_) reference genome (Paterson *et al.* 2012) and the *Gossypium arboreum* (A_2_) reference genome (Li *et al.* 2014). Alignments were corrected to report SNP positions.

### SNP screening

A set of 48 randomly selected intraspecific SNP markers were selected for experimental screening to estimate average validation rate of *in silico* determined intraspecific SNPs. Primers were designed using previously mentioned parameters in BatchPrimer3, and KASP assays were run per manufacturer instructions. A “*G. hirsutum* screening panel” that included 32 *G. hirsutum* lines, *G. barbadense* line 3-79, and water nontemplate controls was used (Table S2). An additional set of 96 markers developed from the 3-79 line were selected based on diploid (D_5_ genome) alignment information with inferred positioning on allotetraploid chromosomes 12 and 26 (Blenda *et al.* 2012). The markers were designed and screened on the *G. barbadense* screening panel (Table S1).

A random set of 32 markers developed from *G. longicalyx* were selected and primers were designed. KASP assays were run per manufacturer’s instructions on a “*G. longicalyx* screening panel” that contained *G. longicalyx* (×2), *G. hirsutum* cv. TM-1 (×2), synthetic allotetraploid “FADD” (×2), 2 BC1F1 samples (FADD × TM-1), *G. barbadense* line 3-79, *G. hirsutum* accession TX0231, and two nontemplate controls (Table S3). Plates were cycled and analyzed as previously mentioned. SNP sequences and primers for all screened markers are listed in Table S4.

### Interspecific linkage mapping

Good markers obtained in screening of the *G. barbadense* SNPs were used to genotype 118 F2 (3-79×TM-1) individuals, two parents (*G. hirsutum* line TM-1 and *G. barbadense* line 3-79), and F1 (3-79×TM-1) as controls. KASP assays were run using the Fluidigm system in 96.96 dynamic array format, utilizing multiple arrays, and read using the Fluidigm BioMark HD (Fluidigm, San Francisco, CA). Clustering for genotyping was performed using Fluidigm SNP Genotyping Analysis software. Genotypes of the 118 F2s for successfully genotyped markers were imported into JoinMap 4.1 (Van Ooijen 2011), and identical markers were removed (Table S5) and linkage mapped using the maximum likelihood algorithm and Haldane’s mapping function with default parameters. Linkage groups were determined using LOD scores of 5.0 or more. Cytogenetic stocks including F1 hypo-aneuploids, each deficient for a known *G. hirsutum* chromosome, were also genotyped for the same markers.

### Phylogenetic analysis

The VCF file produced from GATK that included genotypes from all 14 samples was imported into R (R Development Core Team 2010). The SNPRelate package (Zheng *et al.* 2012) was used to perform a principle component analysis, and eigenvalue1 and eigenvalue2 were used to visualize samples. A distance matrix was determined between samples and then used for hierarchical clustering over 10,000 permutations (z.threshold = 15, outlier.n = 2). The clustering results were used to create a dendrogram tree of the 14 samples.

## Results

### Candidate SNPs derived from BAC-end sequences

**Figure 1:**
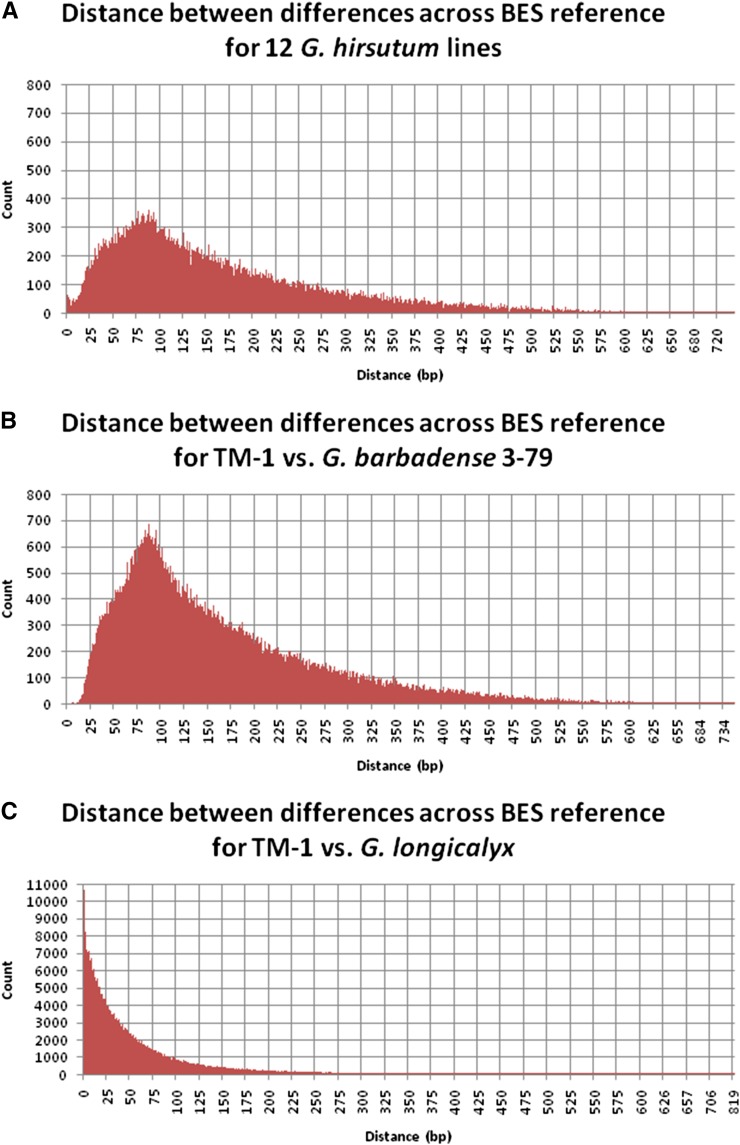
Distance between polymorphisms developed in (A) *G. hirsutum*, (B) *G. barbadense*, and (C) *G. longicalyx* relative to the *G. hirsutum*-derived BAC-end sequences.

**Figure 2:**
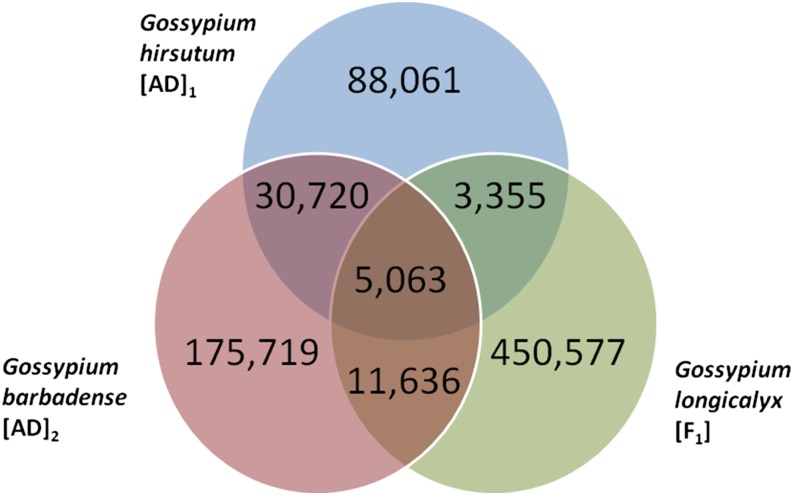
Overlap of SNPs identified using *G. hirsutum*, *G. barbadense*, and *G. longicalyx* samples.

We identified a total of 132,262 intraspecific SNPs for *G. hirsutum* that occurred, on average, every 888 base pairs (bp). The distribution of base pairs between adjacent intraspecific SNPs found on the same BES is, on average, 76 bp (Figure 1A). Interspecific SNPs for *G. barbadense* and *G. longicalyx* occurred at a much higher rate than intraspecific SNPs, which was expected due to longer divergence time between the species. A total of 223,138 interspecific SNPs between *G. barbadense* and *G. hirsutum* were determined. Although SNPs were similarly spaced (78 bp) as the intraspecific SNPs, a larger number of SNPs was identified compared to intraspecific SNPs because they occurred on more BESs (Figure 1B). A total of 470,631 interspecific SNPs were identified between *G. longicalyx* and *G. hirsutum*. The distance distribution between adjacent SNPs found on the same BES from *G. longicalyx* is quite different from the *G. hirsutum* and *G. barbadense* distributions, and it shows that SNPs are more likely to be in close proximity if found on the same BES due to an overall elevated number of SNPs (Figure 1C). This difference is expected and reflects the greater divergence of *G. longicalyx*. Overlap of SNPs identified in multiple species is shown in Figure 2. Considering all identified polymorphisms across all three species, a SNP was identified, on average, every 152 bases and nucleotide diversity across all polymorphic sites or Nei’s Pi was found to be 0.1789.

Types of SNPs were distributed similarly in all three species, with transitions being more abundant than transversions (Figure S1). However, *G. longicalyx* had a lower ratio of transition to transversion (1.63) compared to *G. hirsutum* (2.19) and *G. barbadense* (2.21) (Table 2). The amounts of missing data and heterozygous loci were also similar between species (Table 3). The percentage of missing loci was related to evolutionary divergence between the samples analyzed and the reference, with *G. longicalyx*, *G. barbadense*, and wild *G. hirsutum* line TX0231 exhibiting the largest percentages of missing data (in decreasing order). High levels of missing data in *G. longicalyx* were expected because as a diploid, it would not contain loci from both A- and D-subgenomes of the tetraploid species, but rather would contain mostly only A-subgenome-like loci because it is closely related to the A-genome diploids (Phillips and Strickland 1966). Homozygous differences, which are positions for which two samples are both homozygous for a different base at a single locus, between samples were counted for each pair of samples (Table 4). Overall the number of differences between pairs of cultivated *G. hirsutum* samples was quite variable, ranging from 5562 (Paymaster HS-26 *vs.* Fibermax 832) to 29,052 (TM-1 *vs.* Tamcot Sphinx). Differences between cultivated samples and the uncultivated *G. hirsutum* TX0231 were, in general, quite similar, as was the case between all *G. hirsutum* samples with *G. barbadense* and *G. longicalyx*.

**Table 2:** Distribution of SNP types identified in *G. hirsutum*, *G. barbadense*, and *G. longicalyx*

	*G. hirsutum*		*G. barbadense*		*G. longicalyx*		Overall	
**Total SNP**	132,262	100.0%	187,355	100.0%	450,577	100.0%	770,194	100.0%
**M (A/C)**	11,146	8.4%	16,558	8.8%	42,802	9.5%	70,506	9.2%
**R (A/G)**	45,444	34.4%	64,420	34.4%	139,410	30.9%	249,274	32.4%
**W (A/T)**	11,992	9.1%	16,021	8.6%	60,070	13.3%	88,083	11.4%
**S (C/G)**	6862	5.2%	9188	4.9%	25,420	5.6%	41,470	5.4%
**Y (C/T)**	45,408	34.3%	64,574	34.5%	139,620	31.0%	249,602	32.4%
**K (G/T)**	11,410	8.6%	16,594	8.9%	43,255	9.6%	71,259	9.3%
**Total transition**	90,852	68.7%	128,994	68.9%	279,030	61.9%	498,876	64.8%
**Total transversion**	41,410	31.3%	58,361	31.1%	171,547	38.1%	271,318	35.2%

**Table 3:** Description of missing data and heterozygous loci in the final VCF file for *G. hirsutum*, *G. barbadense*, and *G. longicalyx* samples

Species	Sample	Genotyping Data			
		Missing (No.)	Missing (%)	Heterozygous (No.)	Heterozygous (%)
*Gossypium hirsutum*	TM-1	2144	0.28%	16,880	2.20%
	Sealand 542	4180	0.54%	22,460	2.93%
	PD-1	4435	0.58%	27,039	3.53%
	Paymaster HS-26	4450	0.58%	38,201	4.99%
	M-240 RNR	4146	0.54%	30,450	3.97%
	Fibermax 832	4994	0.65%	31,862	4.16%
	Coker 312	4604	0.60%	21,831	2.85%
	SureGrow 747	4187	0.54%	21,589	2.82%
	Stoneville 474	4058	0.53%	21,554	2.81%
	Tamcot Sphinx	5934	0.77%	22,342	2.92%
	Acala Maxxa	5159	0.67%	20,207	2.64%
	TX0231	12,057	1.57%	24,974	3.29%
*Gossypium barbadense*	3-79	43,530	5.65%	20,443	2.81%
*Gossypium longicalyx*	F1-1	221,786	28.80%	30,204	5.51%

**Table 4:** Pairwise comparison of homozygous different genotypes between 14 resequenced samples using BCFtools command gtcheck

	*G. hirsutum*													
	TM-1	Sealand 542	PD-1	Paymaster HS-26	M-240 RNR	Fibermax 832	Coker 312	SureGrow 747	Stoneville 474	Tamcot Sphinx	Acala Maxxa	TX0231	*G. barbadense* 3-79	*G. longicalyx* F1-1
TM-1	—	15,755	20,141	16,177	17,983	18,405	19,418	18,837	16,103	29,052	25,710	88,174	232,669	444,358
Sealand 542	15,755	—	11,965	9062	10,216	11,711	12,283	13,938	11,275	21,305	17,917	94,229	231,726	442,608
PD-1	20,141	11,965	—	9113	11,672	13,319	13,163	14,435	13,187	20,015	18,488	92,151	229,962	442,294
Paymaster HS-26	16,177	9062	9113	—	6937	5562	7974	9948	8825	13,096	8821	85,791	224,848	440,842
M-240 RNR	17,983	10,216	11,672	6937	—	8865	13,599	12,779	10,929	17,726	16,283	89,815	228,074	442,033
Fibermax 832	18,405	11,711	13,319	5562	8,865	—	13,462	13,507	11,668	15,290	14,310	89,910	226,029	441,551
Coker 312	19,418	12,283	13,163	7974	13,599	13,462	—	12,834	11,891	21,663	18,365	94,397	231,818	442,483
SureGrow 747	18,837	13,938	14,435	9948	12,779	13,507	12,834	—	7046	21,919	17,913	94,752	232,196	443,104
Stoneville 474	16,103	11,275	13,187	8825	10,929	11,668	11,891	7046	—	22,235	17,739	94,936	232,169	442,949
Tamcot Sphinx	29,052	21,305	20,015	13,096	17,726	15,290	21,663	21,919	22,235	—	20,078	98,814	228,004	442,364
Acala Maxxa	25,710	17,917	18,488	8821	16,283	14,310	18,365	17,913	17,739	20,078	—	94,015	231,328	445,417
TX0231	88,174	94,229	92,151	85,791	89,815	89,910	94,397	94,752	94,936	98,814	94,015	—	230,410	442,605
3-79	232,669	231,726	229,962	224,848	228,074	226,029	231,818	232,196	232,169	228,004	231,328	230,410	—	429,140
F1-1	444,358	442,608	442,294	440,842	442,033	441,551	442,483	443,104	442,949	442,364	445,417	442,605	429,140	—

To investigate the functional characteristics of the identified SNPs, all 179,209 BESs used as a reference were first repeat masked, which resulted in a total of 13 million masked bases (11% of total BES length). The masked bases were largely composed of interspersed repeats and retroelements. The remaining bases were aligned to the Genbank nonredundant protein database, which resulted in 9.48% of all BESs having a hit to coding sequences. When all determined SNPs were considered, a similar proportion of genic SNPs (∼7%) was identified in *G. hirsutum* and *G. barbadense*, whereas a larger proportion was identified in *G. longicalyx* (Table 5).

**Table 5:** Annotation of BAC-derived SNPs

Species	BES-derived SNPs Identified				
	Genic		Nongenic		Overall
	No.	%	No.	%	
*G. hirsutum*	10,329	7.81%	121,933	92.19%	132,262
*G. barbadense*	13,274	7.08%	174,081	92.92%	187,355
*G. longicalyx*	97,366	21.61%	353,211	78.39%	450,577
Total	120,969	15.71%	649,225	84.29%	770,194

### Conversion of *in silico* SNPs to assays

*G. barbadense* SNPs developed *in silico* were randomly and nonrandomly (based on theoretical location on chromosomes 12 and 26 based on alignment to the D_5_ sequence) selected for primer design and experimental screening. Initially, a random set of 32 *G. barbadense* markers was selected to determine optimal parameters for homeo-SNP removal. Screening produced a total of 13 SNPs (40.6%) that were found to have acceptable clustering patterns, of which seven (53.8%) were associated and six (46.2%) were not associated with the transcriptome, respectively (Table 1). Thus, no difference was seen for selecting markers based on association with the transcriptome. When attempting to determine the best parameters for *in silico* SNP calling, results from Iteration-1 and Iteration-2 were compared to determine which iteration resulted in the set of markers with the highest overall validation rate. Taking into account the 13 good markers, Iteration-1 produced a success rate of 61.1% (11/18), whereas Interation-2 produced a success rate of 84.6% (11/13); therefore, Iteration-2 mapping parameters (0.99 length fraction and 0.98 similarity fraction) were utilized for final mapping of all samples, except for *G. longicalyx*, as noted in the *Materials and Methods* (Table S6).

Utilizing the SNPs identified in *G. barbadense*, following homeo-SNP removal, an additional 96 interspecific SNPs with successfully designed primers were selected for screening based on inferred positioning on chromosomes 12 and 26. A total of 77 (or 80.2%) markers were categorized as good markers (Table S7).

A total of 48 intraspecific SNP markers were screened on 32 *G. hirsutum* samples and *G. barbadense* line 3-79, which produced 40 good markers or an 83.3% success rate (Table S8). To obtain primers for the 48 markers, 87 SNPs were randomly selected for primer design because primer design was successful in 56% of cases. Sample call rates ranged from 0.175 to 1.000. Excluding the two lowest samples, all samples had greater than 72.5% call rate. Marker call rates ranged from 0.545 to 0.970.

A total of 32 *G. longicalyx* interspecific SNP markers amenable to successful primer design were selected for screening. Primer design for this diploid species was significantly more difficult compared to the other species due to the elevated number of SNPs and their close proximity within a BES (Figure 1C). On screening, a total of 31 (96.9%) SNPs were determined to produce good assays (Table S9). Two of the markers listed as good but exhibit very close clusters (GH_TBb029G06r260, GH_TBb048K02r139), the proximity of which may lead to difficulties for cluster separation with additional samples. So, conservatively, 29 would be regarded as good markers, *i.e.*, a 90.6% success rate.

### Alignment of markers to diploid cotton genomes

When all markers from all three species identified were aligned to the high-quality *G. raimondii* (D_5_) reference (Paterson *et al.* 2012) genome, 38.8% of markers could be aligned to the genome. This was distinctly different from transcriptome-derived markers reported by Hulse-Kemp *et al.* (2014), of which 75.9% could be aligned to the D_5_ genome. The starkly different percentage of mapped markers is likely due to the higher sequence conservation in genic regions. Markers were also aligned to the *G. arboreum* (A_2_) draft genome (Li *et al.* 2014). The percentages of markers aligning to the A_2_ and D_5_ reference genomes were very similar for *G. hirsutum* (70.6%/40.9%) and *G. barbadense* (69.6%/41.1%), whereas the percentage was very different for *G. longicalyx* (55%/18.4%). A larger fraction of markers that can be aligned to the A_2_ genome is expected for *G. longicalyx* because it is an F-genome diploid that is closely related to A-genome diploids, including *G. arboreum*, and it is relatively distant from the D_5_ species, *G. raimondii* (Wendel *et al.* 2009).

Utilizing the alignment information to both A- and D-diploid reference genomes, markers can theoretically be localized to either the A-subgenome or the D-subgenome in the tetraploid by considering whether the markers align uniquely to either the A_2_ or D_5_. In both *G. hirsutum* and *G. barbadense*, ∼52–53% of markers aligned uniquely to A_2_, so they can be putatively localized to the A-subgenome, whereas ∼22–24% of markers aligned uniquely to D_5_ and can be putatively localized to the D-subgenome. Because the A_2_ is approximately twice the size of the D_5_, it is expected that a larger percentage of markers should be uniquely localized to A_2_ (Hendrix and Stewart 2005). *G. longicalyx* had approximately the same proportion of A-specific markers, but the proportion of D-specific markers is considerably lower (12.6%), which is expected because it is closely related to A-genome diploids.

### Interspecific linkage mapping of candidate SNPs and anchoring of contigs

**Figure 3:**
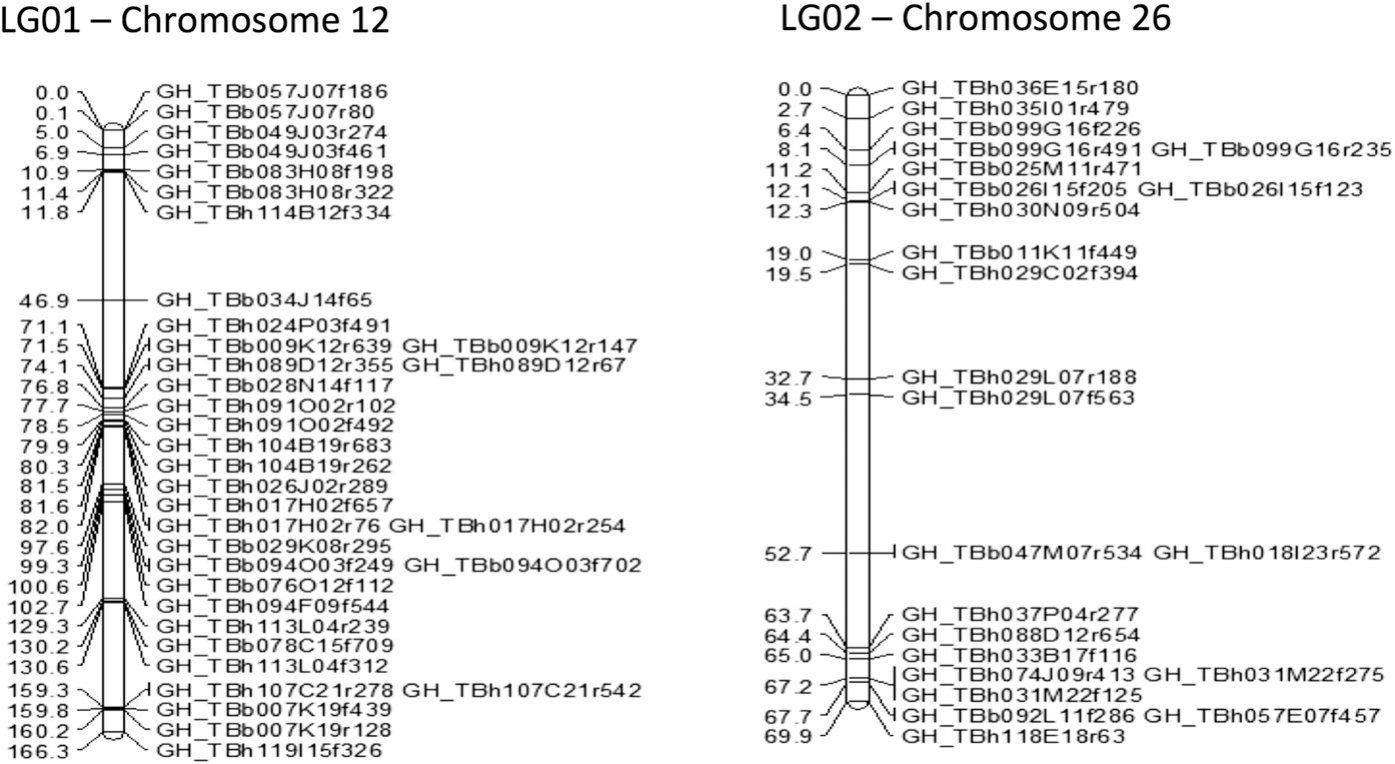
Linkage groups determined utilizing 118 interspecific (*G. barbadense* line 3-79 × *G. hirsutum* line TM-1) F_2_ samples for BAC-end–derived SNPs in JoinMap.

From the random and nonrandom (selected for chromosomes 12 and 26 based on alignment to D_5_ reference sequence) screened SNPs identified from *G. barbadense*, genotypes were obtained for 88 markers that represent 67 unique BAC-end sequences. These 88 markers were screened against a population of 118 F2 (3-79 × TM-1) individuals and F1 hypo-aneuploid stocks. On linkage mapping of the F2s with JoinMap 4.1 software, two linkage groups representing a total of 236.2 cM were obtained (Figure 3). As expected, due to being randomly selected from the entire data set, all of the markers tested from the randomly selected set (11) were not linked and were listed as singletons (Table S10). The resulting two linkage groups were identified as allotetraploid homeologous chromosomes 12 and 26 by loss of heterozygosity in F1 hypo-aneuploid samples and by alignment information to the D_5_ reference genome.

### PCA and dendrogram analysis

**Figure 4:**
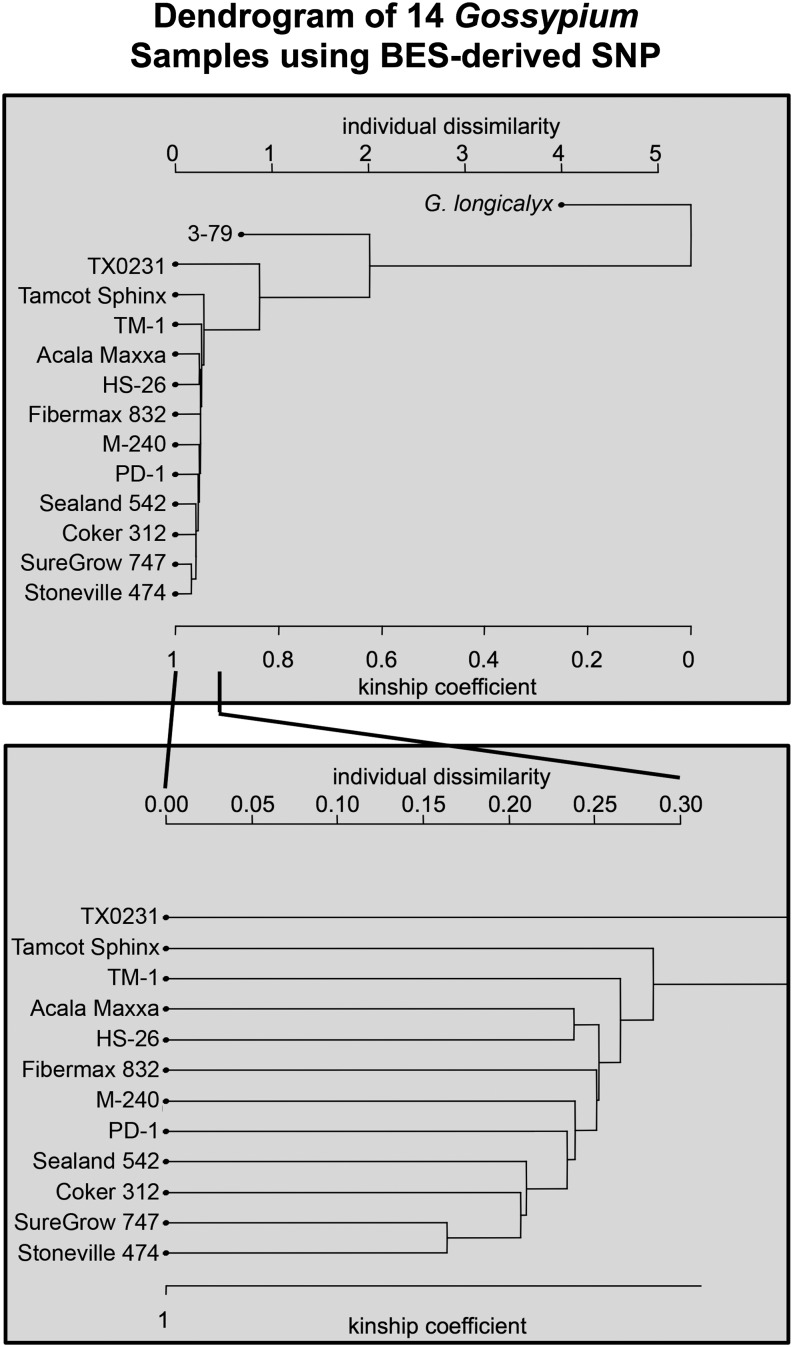
Dendrogram produced by hierarchical clustering utilizing BAC-end sequence-associated SNPs for 12 *G. hirsutum* samples (TM-1, Sealand 542, PD-1, Paymaster HS-26, M-240 RNR, Fibermax 832, Coker 312, SureGrow 747, Stoneville 474, Tamcot Sphinx, Acala Maxxa, TX0231), *G. barbadense* (3-79), and *G. longicalyx* using the SNPRelate package in R.

Principle component analysis with the SNPRelate program was able to successfully separate *G. hirsutum* samples from the other species, *G. barbadense* and *G. longicalyx* (Figure S2). The analysis also showed a slight difference with cultivated *G. hirsutum* lines and the one wild *G. hirsutum* line TX0231. Similarly a dendrogram compiled with the SNPRelate program (Figure 4) was able to separate the wild species and indicated *G. longicalyx* as the out-group, as expected. It also showed the kinship coefficient between *G. hirsutum* samples to be extremely high (near 1) and the individual dissimilarity, or difference between individuals, to be very low (close to 0).

## Discussion

### Reliability of the SNP-based integration of physical and genetic maps

The resulting linkage maps demonstrate the feasibility of integrating physical maps by utilizing markers associated with BAC-end sequences for genetic mapping. In this way, a large number of markers can be identified using resequencing data and can be genetically mapped with a moderately sized mapping population to obtain genetic maps associated with BAC resources. We demonstrate that even with an F2 mapping population of 118 individuals, we are able to obtain recombination events within the length of the BAC (average 120 kilobases) and are able to separate markers associated with the forward and reverse reads of a BAC in some instances. Additionally, multiple markers from the same BES were assayed on the population and linkage mapping places of all those markers except for two from GH_TBh104B19r in the same mapping position. The accuracy of mapping BES-associated SNPs makes it possible to utilize SNP mapping for ordering and orienting BACs and BAC contigs when SNPs are identifiable on both forward and reverse end sequences of single BACs, or when multiple SNPs are identified within a given BES.

### Factors that affect development of BES-associated SNPs in cotton

Development of genomic-based SNPs in cotton has largely been hindered by availability of a high-quality reference, which has led to development efforts largely targeting transcriptome-based SNPs or SNPs identified using reduced representation libraries. In general, genomic enablement for modern cultivated cotton through marker-assisted breeding is constrained by extremely low diversity and large identical regions between the subgenomes. Because of the high similarity between homeologous chromosomes and particularly genic regions, it is extremely difficult to use short next-generation sequencing technology-derived reads to uniquely localize them to a reference sequence. The availability of high-quality BAC-end sequences from a newly developed BAC library resource has provided a high-quality reference of Sanger reads. With a relatively even distribution across the subgenomes, the BESs are a superior reference with very high quality, allowing mapping of short NGS reads obtained from resequencing that have much higher error rates. In this study, this is achieved by using very high stringencies over the entire length of quality-trimmed NGS reads. However, this mapping approach is not feasible with references that are not specific to allotetraploid cotton. When the reference is known to be of much higher quality compared to typical next-generation assemblies, assay development from *in silico*–derived SNPs using this reference is also greatly enhanced. The flanking sequence based on the BES reference should theoretically be correct for TM-1, which can represent cultivated cotton with expectedly low levels of overall diversity and high levels of synteny; this hypothesis holds up to experimental testing as large number of SNPs are able to be successfully genotyped. This is likely due to the automatic inclusion of correct haplotype information for homeo-SNP alleles, which has been shown to increase percentage of co-dominant markers and overall success rates within polyploid cotton (Islam *et al.* 2015).

Although success rates of the BES-associated SNPs are higher than those of previous NGS and RRL approaches (Byers *et al.* 2012; Rai *et al.* 2013; Gore *et al.* 2014; Van Deynze *et al.* 2009; Hulse-Kemp *et al.* 2014; Zhu *et al.* 2014; Islam *et al.* 2015), all *in silico* SNPs were not verified. This could be due to many different reasons, primarily because the BES reference only represents a small portion of the cotton genome, mostly noncoding intergenic regions. Thus, although reads are required to have unique mapping to the reference, it is still possible that additional instances of the sequence exist in the overall cotton genome. This is particularly likely due to ancient paleo-polyploid events that have been discovered in the cotton evolutionary lineage (Paterson *et al.* 2012). Even in largely unique genic regions, gene families and duplicates are common, which will create falsely identified SNPs that, when experimentally assayed, will produce unidentifiable clusters and result in unreliable or bad markers.

### Utilization of BES-associated markers in *G. hirsutum* germplasm

The availability of large numbers of intraspecific SNP markers is essential for marker-assisted breeding in *G. hirsutum* lines, particularly with elite cultivars, which exhibit very low levels of polymorphism. Within a typical elite-by-elite single cross, only a very small percentage of markers will subsequently segregate (typically <5%), so it is difficult to map large numbers of markers from intraspecific crosses. To date, this has led to use of interspecific crosses for the majority of mapping in allotetraploid cotton, such as the one utilized in this effort of *G. hirsutum* (line TM-1) by *G. barbadense* (line 3-79) (Yu *et al.* 2012). However, in this study we see that even within the small set of 48 experimentally tested intraspecific markers (Table S8), most of the 32 tested *G. hirsutum* lines can be uniquely identified. Even the three TM-1 samples obtained from different labs had a small number of identified differences. The KASP assays were largely successful for most samples, except for Fibermax 966 and Deltapine 90, which likely had a much lower DNA concentration when measured via nanodrop, so it was not reaching a genotyping end-point consistently with the other samples and thus led to many uncalled genotypes. Call frequencies for individual markers showed greater variability than sample call frequencies, which is likely due to the need for optimization of PCR conditions for different marker sets. However, when using the Fluidigm system, this is difficult because all marker sets are run under the same conditions. It was found that while SNPs were identified in a relatively small number of samples from U.S. lines, polymorphisms were also transferrable to other germplasm sources, such as the three Australian samples (Delta Opal, Sicot 70, and Siokra 1-4) and one Indian line (MCU-5) included on the panel. This indicates that ascertainment bias may not be very large, and that sample lineage is likely more important than the country of germplasm origin.

Although only small differences exist between lines, the PCA and hierarchical clustering analyses were able to distinguish relationships among the samples. In the PCA, the wild *G. hirsutum* line TX0231 was markedly different from the cultivated samples, and the Tamcot Sphinx (a MAR program–derived sample) was the next most unique sample, which was expected due to the assumed diversity of those samples. Within the cultivated lines, when zoomed-in to a very small section of the plot, the samples appear to occupy three different clusters. Cluster 1 contains TM-1 and Fibermax 832, cluster 2 contains Acala Maxxa, Paymaster HS-26, M-240 RNR, and PD-1, and cluster 3 contains Sealand 542, SureGrow 747, Stoneville 474, and Coker 312. In Fang *et al.* (2013), three of the included samples, SureGrow 747, Stoneville 474, and Fibermax 832, were all identified as being in the same group (group 6). Although we found two of the samples in the same cluster, Fibermax 832 was found in a separate cluster. Fang *et al.* (2013) also identified Acala Maxxa and Paymaster HS-26 to occur in the same group (group 7), which correlates with our analysis where both are in cluster 2. The PCA results correlate with the hierarchical clustering analysis with the shared samples showing the greatest relationship: Acala Maxxa and Paymaster HS-26 from Fang group 7, and SureGrow 747 and Stoneville 474 from Fang group 6. The TM-1 sample, which was the sample utilized as a reference, was also found to have the largest individual dissimilarity after the MAR sample (Tamcot Sphinx). It may be possible that this information is correct because TM-1 was a line established in the mid 20th century (Kohel *et al.* 1970); however, it is also likely that due to ascertainment bias from using this sample as the reference, it looks more different than the other cultivated *G. hirsutum* lines.

### Development of BES-associated markers is helpful for integration of physical and genetic maps

The abundance of markers and accuracy of localization using SNPs associated with BAC-end sequences will be extremely helpful for integrating an allotetraploid cotton physical map with genetic maps. On finalization of an allotetraploid cotton physical map, BES-associated SNPs can also be utilized to integrate unplaced contigs and singletons to enhance the completion of a quality draft reference sequence. Fine- mapping utilizing BES-associated SNPs with a large population can also be used to correct ordering and localization of contigs. Integrating genetic maps with quantitative trait loci (QTL) mapping will allow for utilization of BAC resources for QTL cloning and fine-scale investigation of important regions in the cotton genome.

## Conclusions

The largest set of intraspecific and interspecific SNPs for cultivated cotton to date has been developed. These SNPs are associated with BACs and will serve as an interface between future physical and genetic maps. They were developed using genomic sequences from multiple lines and species aligned to BAC-end sequences generated by Sanger sequencing, which provided a high-quality reference. Experimental validation was highly successful and indicated that the SNPs will allow for future high-density mapping. Furthermore, additional lines can be resequenced and quickly genotyped for the identified SNP positions as shown here using the GATK software. The developed markers complement currently available genic-based SNPs and simple sequence repeat markers, and provide a largely evenly distributed set of markers for mapping the entire cotton genome with high-density. This will promote more extensive genomic-based studies and breeding of cotton.

## 

## Supplementary Material

Supporting Information
