# Supplementary material for: BAC-End Sequence-Based SNP Mining in Allotetraploid Cotton (Gossypium) Utilizing Resequencing Data, Phylogenetic Inferences, and Perspectives for Genetic Mapping
Source: G3 (Bethesda). 2015 Apr 9;5(6):1095–105. doi: 10.1534/g3.115.017749 (PMC4478540; doi:10.1534/g3.115.017749)
Supplement: Supporting Information [file supp_5_6_1095__index.html]

BAC-End Sequence-Based SNP Mining in Allotetraploid Cotton (Gossypium) Utilizing Resequencing Data, Phylogenetic Inferences, and Perspectives for Genetic Mapping — Supporting Information 

# BAC-End Sequence-Based SNP Mining in Allotetraploid Cotton (*Gossypium*) Utilizing Resequencing Data, Phylogenetic Inferences, and Perspectives for Genetic Mapping

## Supporting Information for Hulse-Kemp *et al.*, 2015

**Files in this Data Supplement:**

- Supporting Information - Figures S1-S2 and Tables S1-S10 (PDF, 719 KB)
- Figure S1 - Distribution of SNP types identified *in silico* for *G. hirsutum, G. barbadense, G. longicalyx* and overall by counts (A.) and percentages (B.). (PDF, 639 KB)
- Figure S2 - Principle component analysis utilizing BAC-end sequence derived SNPs for twelve *G. hirsutum* samples (TM-1, Sealand 542, PD-1, Paymaster HS-26, M-240 RNR, Fibermax 832, Coker 312, SureGrow 747, Stoneville 474, Tamcot Sphinx, Acala Maxxa, TX0231), *G. barbadense* (3-79), and *G. longicalyx* using the SNPRelate package in R. (PDF, 558 KB)
- Table S1 - Layout of *G. barbadense* screening panel for testing KASP assays. (.xlsx, 9 KB)
- Table S2 - Layout of *G. hirsutum* screening panel for testing KASP assays. All samples are *G. hirsutum* lines except for sample noted GB, which is *G. barbadense*. \*WAR-Stelly Lab line and TAMU-Zhang Lab line. (.xlsx, 10 KB)
- Table S3 - Layout of *G. longicalyx* screening panel for testing KASP assays. (.xlsx, 9 KB)
- Table S4 - SNP with flanking sequences and primer sequences for common and allele-specific primers used to assay SNPs. (.xlsx, 29 KB)
- Table S5 - Markers that yielded identical genotyping patterns (Identical markers) in linkage groups 12 (LG01) and 26 (LG02), as determined with JoinMap4.1. (.xlsx, 9 KB)
- Table S6 - Sequencing and mapping statistics for analyzed samples. (.xlsx, 12 KB)
- Table S7 - KASP assay screening results for *G. barbadense* derived markers screened on a *G. barbadense* screening panel. (.xlsx, 11 KB)
- Table S8 - KASP assay screening results for *G. hirsutum* derived markers screened on a *G. hirsutum* screening panel and sample panel genotypes. (.xlsx, 18 KB)
- Table S9 - KASP assay screening results for *G. longicalyx* derived markers screened on a *G. longicalyx* screening panel and sample panel genotypes. (.xlsx, 11 KB)
- Table S10 - List of markers not integrated into a linkage group via JoinMap 4.1. (.xlsx, 8 KB)
