# Supplementary material for: BAC-End Sequence-Based SNP Mining in Allotetraploid Cotton (Gossypium) Utilizing Resequencing Data, Phylogenetic Inferences, and Perspectives for Genetic Mapping
Source: G3 (Bethesda). 2015 Apr 9;5(6):1095–105. doi: 10.1534/g3.115.017749 (PMC4478540; doi:10.1534/g3.115.017749)
Supplement: Supporting Information [file supp_g3.115.017749_017749SI.pdf]

G3: Genes | Genomes | Genetics

BAC-End Sequence-Based SNP Mining in Allotetraploid Cotton (*Gossypium*) Utilizing Resequencing Data, Phylogenetic Inferences and Perspectives for Genetic Mapping

Amanda M. Hulse-Kemp[*,§], Hamid Ashrafi [†], Kevin Stoffel[†], Xiuting Zheng[*], Christopher Saski[‡], Brian E. Scheffler[**], David D. Fang[§§], Z. Jeffrey Chen[††], Allen Van Deynze[†], and David M. Stelly[*,§,‡‡]

[*]Department of Soil and Crop Sciences, Texas A&M University, College Station, TX, USA

[§]Interdisciplinary Genetics Program, Texas A&M University, College Station, TX, USA

[†]Seed Biotechnology Center, University of California, Davis, CA, USA

[‡]Clemson University Genomics Institute, Clemson University, 51 New Cherry Street, Clemson, SC, USA

[**]USDA-ARS, Genomics and Bioinformatics Research Unit, 141 Experiment Station Rd., Stoneville, MS, USA

[§§]USDA-ARS, Cotton Fiber Bioscience Research Unit, 1100 Robert E. Lee Blvd., New Orleans, LA, USA

[††]Department of Molecular Biosciences, Center for Computational Biology and Bioinformatics, and Institute for Cellular and Molecular Biology, The University of Texas, Austin, TX, USA

[‡‡]Corresponding author: DMS (stelly@tamu.edu)

Mailing Address: Texas A&M University, 370 Olsen Blvd. 2474 TAMU, College Station, TX 77843-2474

DOI: 10.1534/g3.115.017749

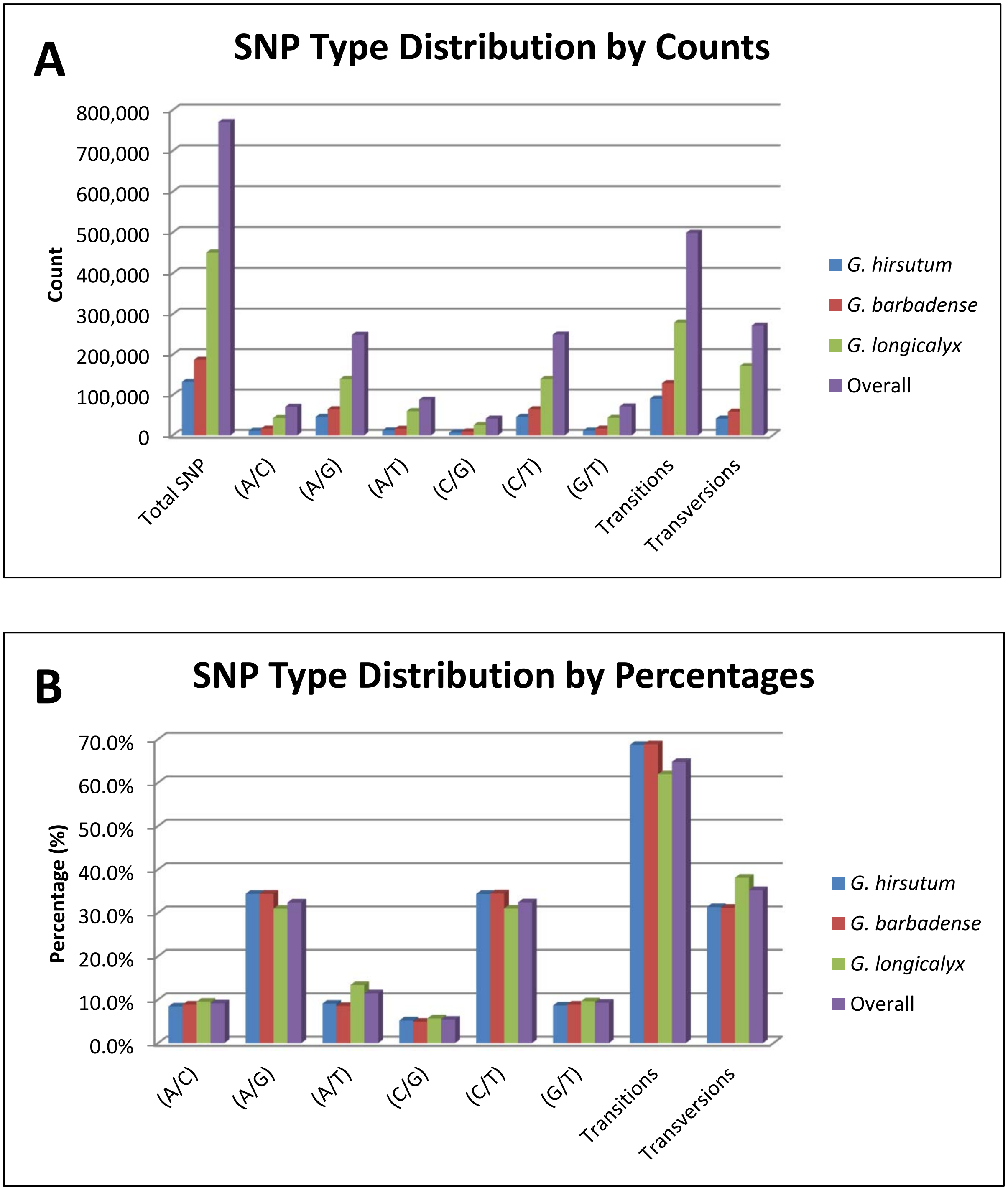

Figure S1 Distribution of SNP types identified *in silico* for *G. hirsutum*, *G. barbadense*, *G. longicalyx* and overall by counts (A.) and percentages (B.).

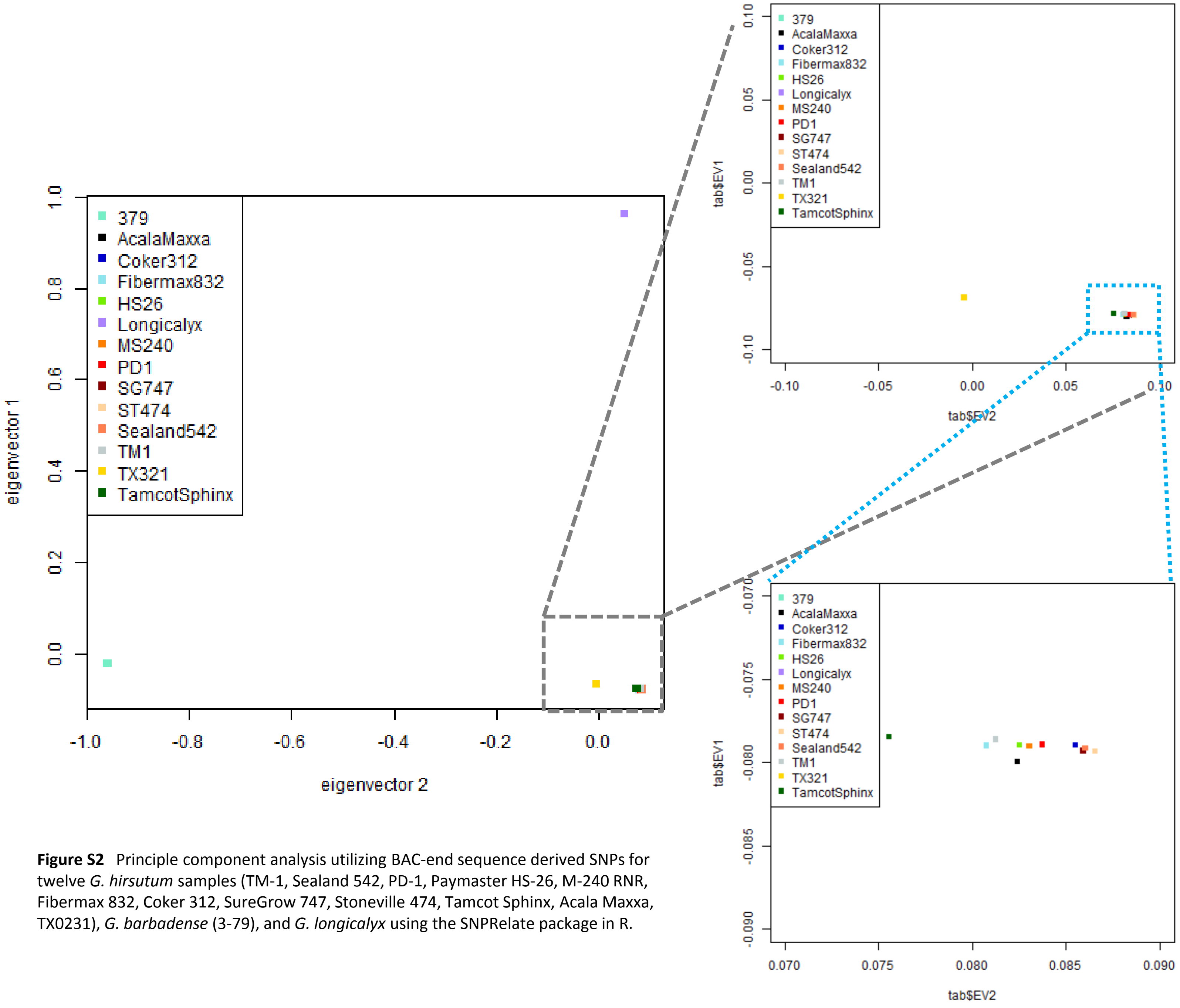

Figure S2 Principle component analysis utilizing BAC-end sequence derived SNPs for twelve *G. hirsutum* samples (TM-1, Sealand 542, PD-1, Paymaster HS-26, M-240 RNR, Fibermax 832, Coker 312, SureGrow 747, Stoneville 474, Tamcot Sphinx, Acala Maxxa, TX0231), *G. barbadense* (3-79), and *G. longicalyx* using the SNPRelate package in R.

Tables S1-S10
Available for download as Excel files at www.g3journal.org/lookup/suppl/doi:10.1534/g3.115.017749/-/DC1

Table S1. Layout of *G. barbadense* screening panel for testing KASP assays.

Table S2. Layout of *G. hirsutum* screening panel for testing KASP assays. All samples are *G. hirsutum* lines except for sample noted GB, which is *G. barbadense*. *WAR-Stelly Lab line and TAMU-Zhang Lab line.

Table S3. Layout of *G. longicalyx* screening panel for testing KASP assays.

Table S4. SNP with flanking sequences and primer sequences for common and allele-specific primers used to assay SNPs.

Table S5. Markers that yielded identical genotyping patterns (Identical markers) in linkage groups 12 (LG01) and 26 (LG02), as determined with JoinMap4.1.

Table S6. Sequencing and mapping statistics for analyzed samples.

Table S7. KASP assay screening results for *G. barbadense* derived markers screened on a *G. barbadense* screening panel.

Table S8. KASP assay screening results for *G. hirsutum* derived markers screened on a *G. hirsutum* screening panel and sample panel genotypes.

Table S9. KASP assay screening results for *G. longicalyx* derived markers screened on a *G. longicalyx* screening panel and sample panel genotypes.

Table S10. List of markers not integrated into a linkage group via JoinMap 4.1.
