# Supplementary material for: BAC-End Sequence-Based SNP Mining in Allotetraploid Cotton (Gossypium) Utilizing Resequencing Data, Phylogenetic Inferences, and Perspectives for Genetic Mapping
Source: G3 (Bethesda). 2015 Apr 9;5(6):1095–105. doi: 10.1534/g3.115.017749 (PMC4478540; doi:10.1534/g3.115.017749)
Supplement: Supporting Information [file supp_g3.115.017749_FigureS1.pdf]

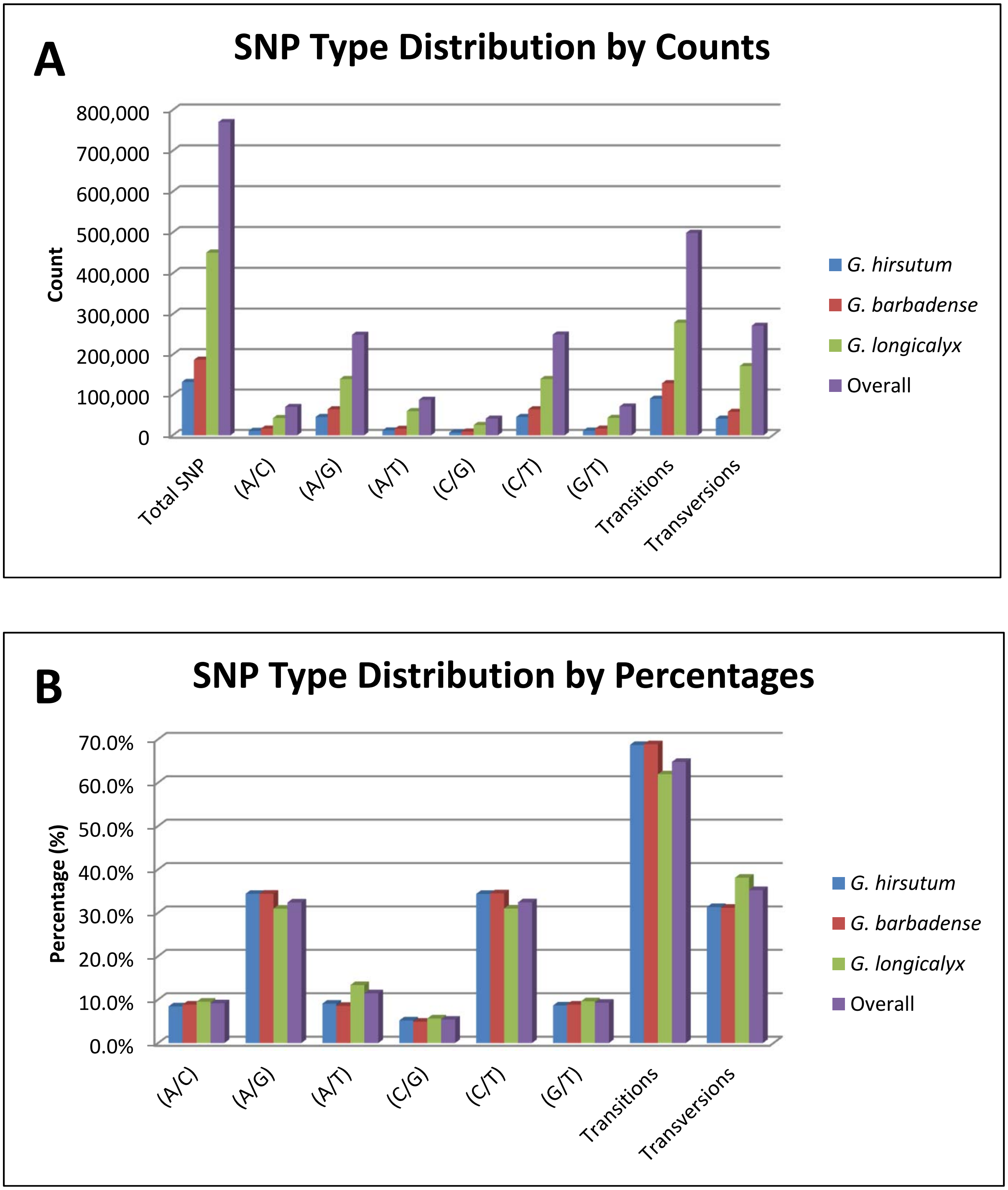

Figure S1 Distribution of SNP types identified *in silico* for *G. hirsutum*, *G. barbadense*, *G. longicalyx* and overall by counts (A.) and percentages (B.).
