# Supplementary figures and images for: BAC-End Sequence-Based SNP Mining in Allotetraploid Cotton (Gossypium) Utilizing Resequencing Data, Phylogenetic Inferences, and Perspectives for Genetic Mapping
Source: G3 (Bethesda). 2015 Apr 9;5(6):1095–105. doi: 10.1534/g3.115.017749 (PMC4478540; doi:10.1534/g3.115.017749)

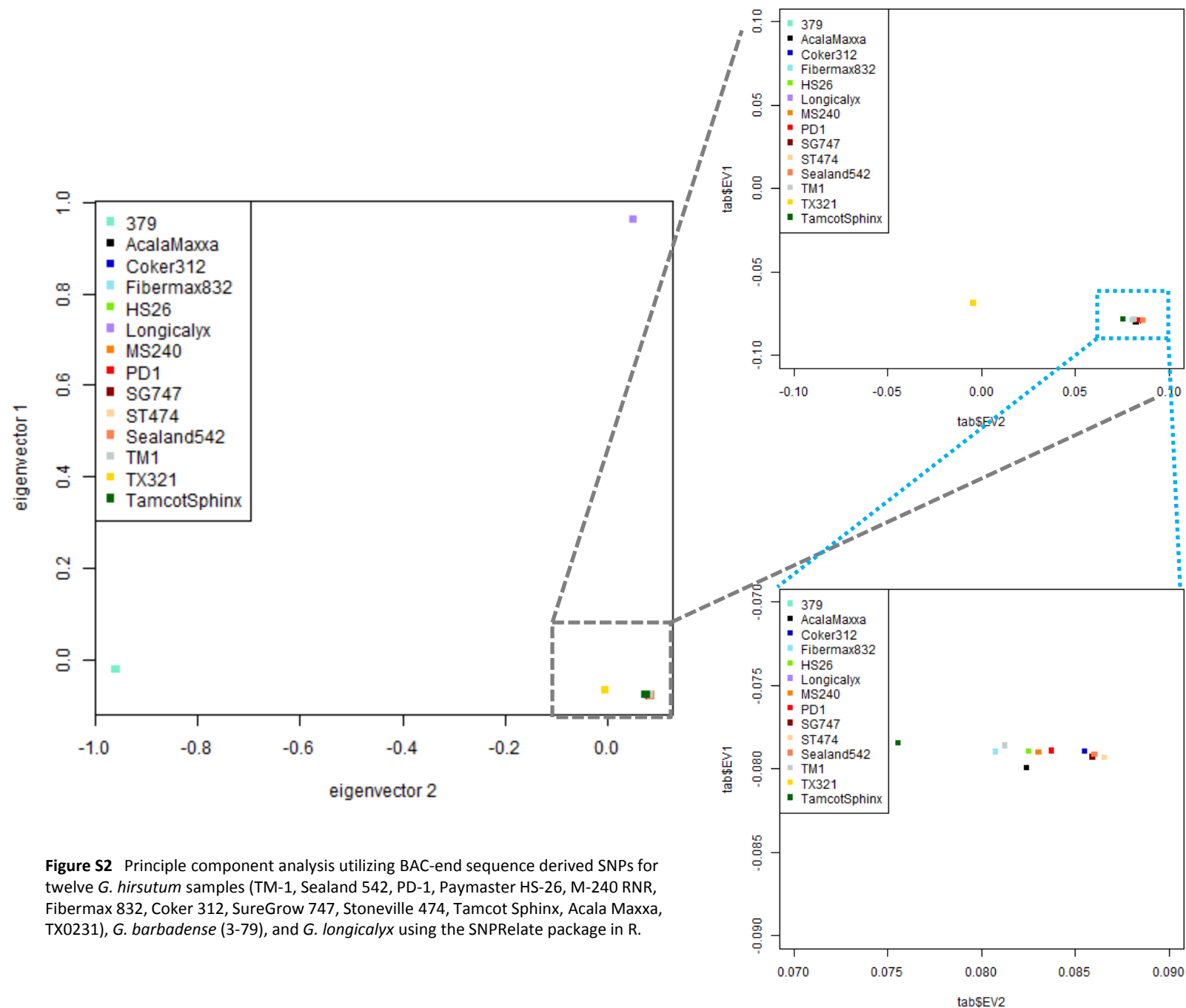

Supplement: Supporting Information [file supp_g3.115.017749_FigureS2.pdf]
